# Gene therapy for glaucoma: Targeting key mechanisms

**DOI:** 10.1016/j.visres.2024.108502

**Published:** 2024-12

**Authors:** Jeff Henderson, Jeffrey O’Callaghan, Matthew Campbell

**Affiliations:** Smurfit Institute of Genetics, Trinity College Dublin, Dublin 2, Ireland

**Keywords:** Gene therapy, Glaucoma, Adeno-associated virus

## Abstract

Glaucoma is a group of optic neuropathies characterised by progressive retinal ganglion cell (RGC) degeneration and is the leading cause of irreversible blindness worldwide. Current treatments for glaucoma focus on reducing intraocular pressure (IOP) with topical medications. However, many patients do not achieve sufficient IOP reductions with such treatments. Patient compliance to dosing schedules also poses a significant challenge, further limiting their effectiveness. While surgical options exist for resistant cases, these are invasive and carry risks of complications. Thus, there is a critical need for better strategies to prevent irreversible vision loss in glaucoma. Gene therapy holds significant promise in this regard, offering potential long-term solutions by targeting the disease’s underlying causes at a molecular level. Gene therapy strategies for glaucoma primarily target the two key hallmarks of the disease: elevated IOP and RGC death. This review explores key mechanisms underlying these hallmarks and discusses the current state of gene therapies targeting them. In terms of IOP reduction, this review covers strategies aimed at enhancing extracellular matrix turnover in the conventional outflow pathway, targeting fibrosis, regulating aqueous humor production, and targeting myocilin for gene-specific therapy. Neuroprotective strategies explored include targeting neurotrophic factors and their receptors, reducing oxidative stress and mitochondrial dysfunction, and preventing Wallerian degeneration. This review also briefly highlights key research priorities for advancing gene therapies for glaucoma through the clinical pipeline, such as refining delivery vectors and improving transgene regulation. Addressing these priorities will be essential for translating advancements from preclinical models into effective clinical therapies for glaucoma.

## 1 Introduction

### 1.1 Glaucoma: An overview

Glaucoma is a group of optic neuropathies characterised by progressive optic nerve and retinal ganglion cell (RGC) degeneration. Affecting upwards of 80 million people, glaucoma is the leading cause of irreversible blindness worldwide (Tham et al., 2014). While the pathogenesis of glaucoma is not fully understood, risk factors include age, family history, and elevated intraocular pressure (IOP) (Weinreb, Aung, and Medeiros, 2014). The normal IOP range in adults is 10–21 mmHg, with IOP values exceeding 21 mmHg being classified as hypertensive (Khaw, Shah, and Elkington, 2004). Glaucoma can be broadly classed into open-angle glaucoma (OAG) and angle-closure glaucoma (ACG). These forms are distinguished based on the state of the angle between the iris and the cornea (the irideocorneal angle). This is the site of aqueous humour (AH) drainage, which, when obstructed, leads to increases in IOP. In OAG, drainage is restricted despite the irideocorneal angle remaining open. Contrastingly in ACG, elevated intraocular pressure occurs as a direct result of the iris becoming displaced and obstructing the irideocorneal angle. Glaucoma can be further broken down into primary and secondary forms. In primary glaucoma, elevations in IOP are idiopathic, while in secondary glaucoma, ocular hypertension is caused by known factors such as systemic disease, trauma, or treatment with corticosteroids (Weinreb, Aung, and Medeiros, 2014). Primary open angle glaucoma (POAG) is the most prevalent subtype of glaucoma, accounting for 74 % of all glaucoma cases (Quigley and Broman, 2006).

As it is the only modifiable risk factor for the disease, current treatments for glaucoma focus on reducing intraocular pressure (IOP) with topically administered pressure-reducing medications. However, between 25 % and 50 % of patients do not achieve sufficient IOP reductions with first-line prostaglandin analogues, and up to 10 % of patients (estimated at 6.4 million people globally) respond sub optimally to polypharmacy eye-drop treatments (Noecker et al., 2003, Scherer, 2002).

Despite the effectiveness of multiple topical drops when used correctly and the development of new IOP lowering medications, such as the Rho Kinase inhibitor Netarsudil (Khouri et al., 2019), adherence to dosing schedules poses a significant challenge, resulting in poor compliance that limits the real-world effectiveness of these treatments (Newman-Casey et al., 2015, Okeke et al., 2009). While surgical options such as minimally invasive glaucoma surgery, trabeculectomy, and drainage tubes are available for resistant cases, these procedures are invasive and carry risks of complications. Additionally, a subset of patients with OAG exhibit normal tension glaucoma (NTG), characterized by IOP values that remain within the statistically normal range without treatment. Epidemiological studies indicate that NTG accounts for approximately 20–40 % of all POAG cases among Caucasians and Africans and between 47 % to 92 % in Asian populations (Salvetat et al., 2023, Klein et al., 1992, Zhao et al., 2019). While lowering of IOP is also beneficial in slowing the progression of NTG, some patients continue to experience disease progression despite IOP reduction (Anderson, 2003). Thus, there is an urgent need for better clinical tools and approaches to prevent irreversible vision loss in glaucoma patients.

### 1.2 Gene therapy for glaucoma

**Fig. 1:**
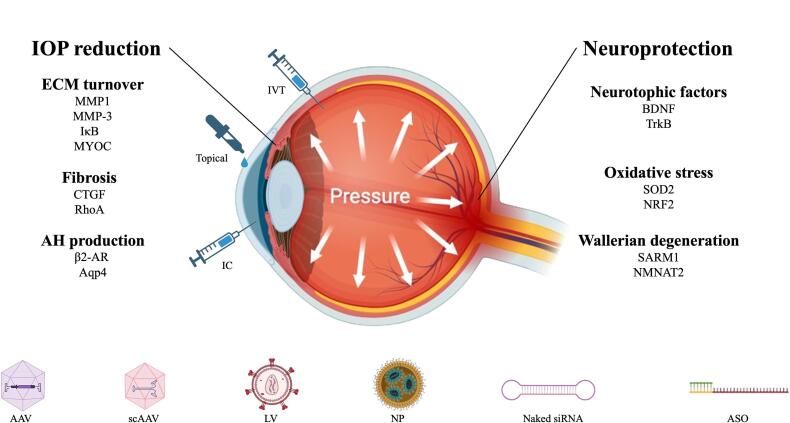
Overview of proposed targets, delivery methods and vectors for glaucoma gene therapy. Gene therapies for glaucoma aim to reduce intraocular pressure (IOP) (left) or protect retinal ganglion cells (right). Approaches to reduce IOP include increasing extracellular matrix (ECM) turnover, reducing fibrosis and increasing aqueous humour (AH). Neuroprotective approaches include increasing levels of neurotrophic factors, reducing oxidative stress, and inhibiting Wallerian degeneration. Delivery methods include intravitreal (IVT), topical, and intracameral (IC). Vectors for gene delivery include adeno-associated viruses (AAV), self-complimentary (sc)AAVs, lentiviruses (LV), nanoparticles (NP), naked small interfering (si)RNA, and antisense aligonucleotides (ASO). Created with Biorender (www.biorender.com)

Adapted from Sulak, Liu, and Smedowski (2024)).

To date, progress towards gene therapies targeting glaucoma has been relatively limited compared to those treating inherited retinal degenerations. This disparity can be partly attributed to the multifactorial nature of glaucoma, with less than 10 % of glaucoma cases exhibiting a monogenic inheritance pattern (Weinreb, Aung, and Medeiros, 2014). Nevertheless, there has been significant progress made in this area in recent years. Gene therapy approaches in glaucoma can be broadly divided into two strategies: reducing IOP and protecting RGCs. Both strategies come with unique challenges relating to the availability of suitable animal models, target cells and pathways, and gene delivery mechanisms (Fig. 1). While neuroprotection offers a distinct advantage by targeting the ultimate cause of sight loss, the retina is a more immunogenic environment compared to the anterior chamber of the eye (Hori, 2008). Thus, in the retina immune responses to viral vectors or their expressed therapeutic proteins can pose complications, requiring careful consideration of immunomodulatory strategies. Progress towards gene therapy for glaucoma has also been limited by the incomplete understanding of its pathophysiology, which is essential for advancing effective treatments. This review will discuss a selection of mechanisms underlying the two major hallmarks of glaucoma: ocular hypertension and RGC degeneration. It will then examine the current state of gene therapies targeting these aspects, highlighting recent progress and ongoing challenges. While this review covers selected targets in detail, it is not intended to be comprehensive. For a broader overview of gene therapy targets for glaucoma, readers are directed to Sulak, Liu, and Smedowski (2024).

## 2 Gene therapies targeting ocular hypertension in glaucoma

### 2.1 Aqueous humour outflow dynamics

**Fig. 2:**
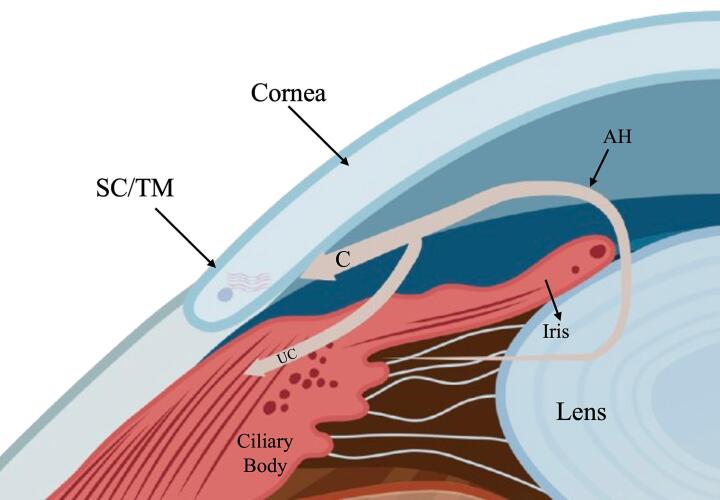
Aqueous humour flow dynamics. Upon secretion from the ciliary body, AH moves from the posterior to the anterior chamber of the eye via the pupil. A temperature gradient causes aqueous humour (AH) to move downwards from the corneal surface, where it is drained via either the conventional outflow pathway (C) or the unconventional outflow pathway (UC). The conventional outflow pathway, involves AH passing through the trabecular meshwork (TM), crossing the inner wall of Schlemm’s canal (SC) into its lumen. Unconventional flow passes through the interstitial spaces between ciliary muscle fibres into the supraciliary and suprachoroidal spaces. Created with Biorender (www.biorender.com)

Adapted from (O’Callaghan, Cassidy, and Humphries 2017).

The flow of AH is also important for providing shape to the globe of the eye via the generation of IOP (To et al., 2002, Goel et al., 2010), while supplying surrounding structures with nutrients, removing waste metabolic products and permitting inflammatory cells and mediators to circulate the eye under pathological conditions. IOP is controlled by the dynamic balance between AH production (inflow) and the resistance to its drainage (outflow) through two key pathways at the iridocorneal angle. The first pathway, the conventional outflow pathway, involves AH passing through the trabecular meshwork (TM), crossing the inner wall of Schlemm’s canal (SC) into its lumen, followed by drainage through collector channels into aqueous veins that ultimately drain into the episcleral venous circulation (Goel et al., 2010). Unconventional flow passes through the interstitial spaces between ciliary muscle fibres and into the supraciliary and suprachoroidal spaces (Fig. 2). From these spaces, AH can either be resorbed by orbital vessels (the uveoscleral pathway) or drained through the vortex veins via the choroid (the uveovortex pathway) (Johnson, McLaren, and Overby, 2017). The conventional outflow pathway is predominant in humans, accounting for approximately 85 % of AH outflow (Bill and Phillips, 1971), which becomes more pronounced with age (Toris et al., 1999).

### 2.2 Targets for IOP based gene therapies

#### 2.2.1 Targeting ECM turnover at the conventional outflow pathway

Homeostatic ECM turnover is crucial for regulating outflow resistance. This turnover can be triggered by factors such as the stretch or distortion of TM cells, often due to elevated IOP (Bradley et al., 2001). Juxtacanalicular (JCT) cells of the TM continuously express various ECM proteins, and these expression profiles can be altered by exposure to environmental stresses, synthetic agents, or laser trabeculoplasty surgeries (Zhao et al., 2004, Vittal et al., 2005, Lo et al., 2003, Izzotti et al., 2011). ECM turnover, driven by proteinases that target ECM components, is essential for maintaining homeostasis, as reflected by the high gene expression levels in these cells (Vittal et al., 2005). Proteinases involved in ECM turnover include zinc-dependent enzymes such as classical matrix metalloproteinases (MMPs), adamalysisns (ADAM and ADAMTS proteins), and those involved in the plasminogen activation pathway (Keller et al., 2009). MMPs are expressed constitutively throughout the TM, with their expression profiles changing in response to different stimuli (Alexander et al., 1991, Oh et al., 2006, Kelley et al., 2007). The expression of proteinases must be tightly regulated to effectively control outflow resistance. For this purpose, tissue inhibitors of metalloproteinases (TIMPs) exhibit a similar expression pattern to MMPs, with the ratio of the two groups of proteins determining the extent of ECM deposition (Tektas and Lütjen-Drecoll, 2009, Overby et al., 2014). The importance of this balance is underscored by various studies finding an imbalanced MMP:TIMP ratio in glaucoma patients relative to controls (Schlötzer-Schrehardt et al., 2003, Rönkkö et al., 2007, Fountoulakis et al., 2013). Furthermore, perfusion of ocular tissues with MMPs or MMP-activating compounds leads to increased outflow facility (Bradley et al., 1998, Pang et al., 2003, Webb et al., 2006, Groef et al., 2013). Given its importance in regulating outflow resistance, the ECM is an appealing target for gene therapies aimed at reducing ocular hypertension in glaucoma (O’Callaghan, Cassidy, and Humphries, 2017). Several emerging therapies have emerged that exert direct or indirect effects on the ECM, a selection of which will be discussed below.

One strategy involves expressing an MMP-1 transgene within the TM under the control of a glucocorticoid-responsive promoter. Corticosteroid treatments can cause ocular hypertension in a significant number of individuals, thus steroid induced expression of MMP-1 within the TM will result in a reduction in collagen type 1, preventing an increase in IOP (Spiga and Borrás, 2010). The feasibility of this approach was initially demonstrated in sheep by intracamerally injecting an Ad vector containing the transgene, where steroid-induced expression of MMP-1 led to a decrease in IOP (Gerometta et al., 2010). The group later demonstrated the same effect upon transferring the transgene cassette to a self-complimentary (sc)AAV2 vector (Borrás, Buie, and Spiga, 2016). Another approach involves intracameral injection of an AAV9 vector containing a plasmid expressing codon-optimised MMP-3 to transduce the corneal endothelial cells (O’Callaghan et al., 2017, O’Callaghan et al., 2023). As MMP-3 is a secretory protein, its expression in corneal endothelial cells leads to its apical secretion into the AH, where it can reach the outflow tissues via the normal flow patterns of AH. Here, MMP-3 is autoactivated with initial cleaveage by nonspecific proteases and other MMP molecules. This leads to reduced collagen and laminin levels, constriction of actin filaments and disorganisation of fibronectin networks, ultimately increasing outflow facility and reducing IOP. This approach increases outflow facility in two mouse models of glaucoma (Tg-MYOC^Y437H^ myocilin model and the steroid-induced dexamethasone model), non-human primates (NHPs), and human donor eyes. Critically, ocular inflammation was not detected in NHPs following intracameral injection of AAV. Additionally, MMP-3 was detectable in the anterior segment over 200 days after injection, suggesting that this approach is both durable and clinically translatable (O’Callaghan et al., 2023).

Other approaches have aimed to target expression of upstream regulators of MMPs. For example, the secretion of MMPs is known to depend on NF-κB in macrophages and vascular smooth muscle (Bond et al., 2001, Chase et al., 2002). NF-κB-mediated upregulation of MMPs has also been shown in TM cells and human ciliary muscle cells (Lan et al., 2009, Porter et al., 2012). The IκB kinase serves to regulate the cellular response to inflammatory stimuli. NF-κB is inactive when bound to IκB in the cytoplasm, with phosphorylation of the latter leading to release of NF-κB, which then acts as a nuclear transcription factor, stimulating MMP expression in the trabecular meshwork and ciliary body (Ramkumar et al., 2011). It follows that inhibiting IκB in the outflow tissues may be a useful strategy for reducing IOP. Zeng et al. (2020) utilised hyperbranched cationic glycogen derivative vector to deliver siRNA targeting IκBα intracamerally in normotensive rats, leading to increased MMP-2 expression and a significant reduction in IOP 72 h post injection. However, the reduction in IOP was transient, with levels returning to baseline 5 days post-treatment. In a more recent study, naked IκBα-siRNA was injected intracamerally in Rhesus monkeys following laser photocoagulation to induce chronic ocular hypertension, leading a longer lasting reduction in IOP (28 days) that correlated with increased expression of MMPs-2 and −9 in the TM and ciliary body (Sun et al., 2022).

Tissue plasminogen activator (tPA) is another regulator of MMPs and has been shown to increase MMP-9 and MMP-13 expression after ocular injection. Transfection of ocular tissues using adenovirus with the transgene for tPA, encoded by the PLAT gene, was shown to increase outflow facility in wild type mice with subconjunctival injection of the corticosteroid Triamcinolone Acetonide. Interestingly, the serine protease activity of tPA is not critical for elevated outflow facility, and instead functions as a transcriptional regulator of MMPs in the anterior chamber, as shown by mRNA quantification (Gindina et al., 2021, Gindina et al., 2020).

Similar to other studies, the authors found no change in outflow facility in normotensive mice, suggesting that the intervention does not overcorrect or cause hypotony, adding to the attractiveness of ECM-modulating strategies.

Other groups have investigated the use of micro RNAs (miRNAs) to regulate ECM turnover. These are naturally occurring small non-coding RNAs that regulate biological function at the post-transcriptional level by targeting a network of molecules. Notably, the profiles of miRNAs in aqueous humour have been shown to be altered in glaucoma patients (Drewry et al., 2018, Jayaram et al., 2017). In an initial study, Tan et al. (2020) intracamerally miR-21-5p, a miRNA proposed to regulate ECM in TM cells, to the eyes of C57/BL6 mice, leading to a reduction IOP and increased outflow facility. This was accompanied by an increase in MMP-9 and a decrease in TIMP-3 expression (Tan et al., 2020). In a follow-up study, the authors packaged miR-21-5p in a polydopamine–polyethylenimine nanoparticle vector to counteract the rapid degeneration of the molecule. This was shown to enhance the stability of the genetic molecule with minimal cytotoxicity (Tan et al., 2021).

#### 2.2.2 Targeting fibrosis pathways

The ECM of glaucomatous tissues displays similarities to fibrotic tissue, including an excessive accumulation of ECM components such as α-SMA, progressing to a hardening or scarring of connective tissues (Mutsaers et al., 1997, Wight and Potter-Perigo, 2011). Indeed, the ECM at the JCT in glaucoma displays hallmarks of fibrosis in the form of sheath derived plaques, which are particularly evident in the ageing TM (Keller and Acott, 2013, Tektas and Lütjen-Drecoll, 2009).

Additionally, glaucomatous Schlemm’s canal endothelial cells (SCECs) exhibit a fibrotic phenotype, expressing higher levels of α-SMA, collagen I-α1, and fibronectin, relative to healthy SCECs (Kelly et al., 2021). This has led to the development of various gene therapy-based approaches targeting fibrosis pathways at the conventional outflow tissue. TGF-β2 is a cytokine with an important regulatory role in this regard, inducing the expression of collagens, α-SMA, fibronectin, among others, and is known to be upregulated in glaucoma (Guo et al., 2019). TGF-β2 signalling is mediated by elastic microfibrils, specifically the fibrillin component that is responsible for the elasticity of the ECM, which is required for sequestering and activating TGF-β2 complexes (Han et al., 2011). The induction of extracellular matrix (ECM) cross-linking by TGF-β2 resembles the cross-linking observed in glaucomatous tissues and may contribute to the disease pathogenesis (Wallace et al., 2014, O’Callaghan et al., 2017).

Connective tissue growth factor (CTGF), is a downstream target of TGF-β2 and is proposed to be a mediator of increased outflow resistance in POAG, with overexpression in the mouse eye leading to increases in IOP and optic nerve damage, correlated with induction of TM fibronectin and α-SMA (Junglas et al., 2012, Junglas et al., 2009). In a recent study, Dillinger et al. (2018) proposed a novel therapeutic concept in which CTGF-siRNA would be delivered in layer-by-layer coated nanoparticles, with a final layer of hyaluronic acid (HA). The HA-coating is proposed to allow binding to TM and SC cells via CD44, which the authors demonstrate to be upregulated in the glaucomatous anterior chamber. While CTGF gene silencing by this method has yet to be tested in vivo, this approach is of particular interest as the gene silencing may depend on the expression profile of CTGF itself, which could provide high specificity toward tissue with pathological expression changes. Another downstream target of TGF-β cells is the RhoA/Rho-kinase (ROCK) signalling pathway. Activation of this pathway in TM cells leads to actin filament contraction and polymerisation via phosphorylation of LIM kinase-2 and myosin light chain, leading to contraction of the TM and increased outflow resistance (Sit and Manser, 2011). Upon binding of TGF-β to TGF-β receptor 1 on TM cells, a downstream signalling cascade is triggered so that RhoA-GTP and ROCK are activated. This leads to increased TM contractility, reduced AH drainage and elevated IOP (Pattabiraman, Inoue, and Rao, 2015). Inhibition of ROCK signalling has thus been investigated as a potential therapeutic avenue for ocular hypertension in glaucoma. Intracameral injection of ROCK inhibitor Y-27632 in rabbits has been shown to decrease IOP in a dose dependent manner, with a corresponding increase in outflow facility (Honjo et al., 2001). A recent study investigated the effect of siRNA-mediated knockdown of RhoA on TM fibrosis and IOP. The researchers injected TGF-β intracamerally twice a week into adult Sprague Dawley rats to induce ocular hypertension, while also injecting RhoA-siRNA in select animals. TGF-β increased TM contractile proteins and caused IOP spikes by day 7, with sustained IOP elevation and TM fibrosis being observed by day 35. siRhoA abolished the early IOP rise but not the sustained increase or RGC loss. The researchers concluded that RhoA signaling mediates the early IOP rise due to TM contractility but not the sustained elevation from fibrosis, suggesting that RhoA therapies can manage early IOP spikes but not fibrosis-related increases (Hill et al., 2018).

#### 2.2.3 Targeting aqueous humour production

Most therapeutic interventions for reducing IOP in glaucoma focus on increasing AH outflow. However, targeting AH production is also a potentially viable strategy, since IOP depends on both AH production and outflow. Currently, cycloablation of the ciliary body with cyclodiode lasers is a recognised therapeutic strategy in the management of refractory glaucoma, leading to a significant and sustained reduction in IOP. However, such procedures are associated with significant adverse events, leading to a loss of visual acuity in a subset of patients (Rotchford et al., 2010, Murphy et al., 2003). Additionally, β-blockers, which reduce IOP by reducing AH production at the ciliary body, are among most highly prescribed treatments for glaucoma (Sidjanin et al., 2008). However, these are associated with systemic adverse effects such as brachycardia, hypotension and heart failure (Han et al., 2008). Thus, gene therapy based approaches to reduce AH production may be a viable alternative to these approaches. One such example is Bamosiran, a topical eye drop solution containing siRNA targeting β-Adrenergic Receptor 2 (β2-AR). By targeting the same receptors as β-blockers, this approach held promise to reduce IOP without systemic adverse events. In preclinical studies, Bamosirsan was shown to reduce IOP in normal and hypertensive rabbit eyes, in addition to being safe and well tolerated in non-human primates (Martínez et al., 2014). It later progressed to phase 1 (Moreno-Montañés et al., 2014) and phase 2 (Gonzalez et al., 2016) clinical trials where it was shown to be safe and efficacious in humans. However, this treatment carries the same limitations related to non-compliance as conventional eye drops, as its effect on IOP is transient. Thus, despite these results, Bamosiran was ultimately discontinued as it did not offer significant advantages or better cost-benefit compared to timolol eyedrops. More recent approaches have aimed to produce longer-lasting reductions in AH production. A recent study by Wu et al. (2020) aimed to disrupt aquaporin-4 (Aqp4) expression in the ciliary body using CRISPR-Cas9 gene editing. Aquaporins water-transporting transmembrane proteins that are expressed throughout the human body (Schey et al., 2014). They play a critical role in AH production in the ciliary body, as underscored by the fact that Aqp4 null mice display reduced IOP (Zhang, Vetrivel, and Verkman, 2002). The authors utilised an AAV-Sh10 vector system to deliver two Staphylococcus aureus-derived Cas9 (SaCas9)-compatible gRNA targeting exon 1 of AQP1 intravitreally. This led to a significant decrease in IOP in C57BL/6J mice and two models of glaucoma (corticosteroid- and microbead-induced ocular hypertension), which was maintained over a 3–7 week observation period and prevented RGC loss (Wu et al., 2020, Komáromy, 2020).

#### 2.2.4 Myocillin as a gene-specific gene therapy target

As most cases of glaucoma do not follow a Mendelian inheritance pattern, the majority of gene therapies targeting ocular hypertension are gene-agnostic. However, in cases where glaucoma is caused by a well-defined genetic mutation, gene-specific therapies offer an attractive target due to their precise genetic aetiology. One such example is the gene encoding myocilin (MYOC), which was the first locus to be linked with POAG (Stone et al., 1997). MYOC mutations account for 2–4 % of overall POAG cases and 10–30 % of juvenile-onset cases (Menaa et al., 2011). *MYOC* is highly expressed in trabecular meshwork (TM) cells as well as in various ocular and non-ocular tissues (Karali et al., 2000, Takahashi et al., 1998). However, mutations in MYOC lead to a gain of function phenotype, with mutant MYOC forming detergent insoluble aggregates and accumulating in the endoplasmic reticulum (ER), resulting in ER stress (Liu and Vollrath, 2004, Yam et al., 2007, Joe et al., 2003). Chronic ER stress in TM cells results in cell death, ultimately elevating IOP (Zode et al., 2011, Joe et al., 2003), as modelled in the Tg-MYOCY437H mouse model, transgenic for the human Y437H allele (Zode et al., 2011).

Given that MYOC is not required for IOP regulation under normal conditions and that mutant MYOC leads to a gain of function phenotype, it represents an ideal target for IOP-lowering gene therapy (Kim et al., 2001, Gould et al., 2004). Two recent studies focused on using CRISPR/Cas9 gene editing to alleviate ocular hypertension in MYOC-associated glaucoma. In a proof-of-concept study, MYOC knockout was achieved by delivering a guide RNA (gRNA) targeting exon 1 of *MYOC* and Cas9 endonuclease in an Ad5 vector (Jain et al., 2017). One month post-treatment, Tg-MYOCY437H mice exhibited lower IOP, improved RGC function and reduced expression of ER stress markers. Additionally, mutant MYOC expression was reduced in a human ex vivo anterior segment perfusion culture system, supporting its potential utility in humans (Jain et al., 2017). Although an Ad5 vector was used in this study due to its strong tropism for the TM (Shepard et al., 2010), it is unsuitable for clinical use due to its pro-inflammatory nature (Millar et al., 2023). Indeed, the authors noted that inflammation was observed in the anterior chamber of mice following Ad5 injection (Jain et al., 2017). A follow-up study aimed to address this by investigating the efficacy of AAVs and lentiviral (LV) particles to target the TM (Patil et al., 2024). Of the various vectors and delivery routes assessed, it was observed that intravitreal (IVT) delivery of LV led to the most selective and efficient tropism to the TM. This approach was subsequently used to deliver Cas9 and gRNA targeting *MYOC* in Tg-MYOCY437H mice, leading to decreased accumulation of MYOC and significantly lower IOP following a single injection.

## 3 Neuroprotection based gene therapy strategies

### 3.1 Glaucomatous optic neuropathy

While ocular hypertension is the only modifiable risk factor for glaucoma, the exact mechanism by which elevated IOP leads to RGC degeneration is not fully understood. There are multiple hypothesised mechanisms of RGC death, such as mechanical damage, reduced axonal transport and oxidative stress. These factors likely interact in complex ways and may contribute to differing degrees depending on the individual and the specific subtype of glaucoma. Understanding these mechanisms and their interactions is of critical importance for the development of gene therapies targeting glaucomatous optic neuropathy. The principal site of insult to RGC cells in glaucoma is the laminar region of the optic nerve head (ONH). This can be attributed to the lamina cribrosa (LC), a fenestrated structure composed of collagen fibres that provides structural and nutritional support to RGC axons as they leave the sclera. From a biomechanical standpoint, the LC is a weak point in the posterior wall of the eye, as it is approximately one-third the thickness of the sclera and only comprises around 40 % of the total tissue volume in the laminar region of the ONH. Thus the LC must support the ONH against mechanical strain caused by IOP elevation, while also providing a pathway for RGC axons to leave the eye (Downs and Girkin, 2017). Scanning electron microscopy studies of glaucomatous ONHs show that the first structural evidence of damage is in the backward collapse of the anterior LC, followed by a prominent bowing of the whole structure (Quigley et al., 1981). Ocular hypertension may thus cause compression, deformation, and remodelling of the LC, resulting in mechanical damage to RGC axons and disrupted axonal transport (Weinreb, Aung, and Medeiros, 2014). Animal models of ocular hypertension display reduced anterograde and retrograde axonal transport of neurotrophic factors, indicating that pathological increases in IOP may lead to RGC death via trophic insufficiency (Quigley et al., 2000, Pease et al., 2000). Additionally, simultaneous IOP-induced mitochondrial dysfunction may further exacerbate RGC dysfunction (Ju et al., 2008). It must also be noted that increases in IOP are not the sole cause of glaucomatous RGC death, as evidenced by the existence of normal-tension glaucoma, in which patients develop glaucoma despite presenting with IOPs within the normal range (Lee, Bathija, and Weinreb, 1998). In such cases, an elevated pressure differential across the LC has been proposed as a contributing factor to optic nerve damage, which may occur when orbital cerebrospinal fluid (CSF) pressure is low (Jonas and Wang, 2013).

### 3.2 Targets

#### 3.2.1 Targeting neurotrophic factors and their receptors

Neurotrophins are a family of proteins that support the survival, growth, and differentiation of neurons and include nerve growth factor (NGF), brain derived neurotrophic factor (BDNF), ciliary neurotrophic factor (CNTF), and glial cell line-derived neurotrophic factor (GDNF), among others. Of the neurotrophins, BDNF and its receptor, TrkB, have emerged as potentially important mediators of glaucomatous RGC degeneration. Axonal transport of BDNF is disrupted in rodent and primate models of ocular hypertension (Quigley et al., 2000, Pease et al., 2000). Additionally, there is growing evidence of impaired BDNF signalling in human glaucoma, with reduced levels being observed in the optic nerve head, tear film, and serum of glaucoma patients, relative to controls (Gupta et al., 2014, Ghaffariyeh et al., 2009, Oddone et al., 2017).

Given the potential role of BDNF signalling in glaucoma development, its application as a neuroprotective agent has been explored. Early studies demonstrated that intravitreal injections of recombinant BDNF could delay RGC death in a rat hypertension model and an optic nerve crush model in cats (Ko et al., 2001, Chen and Weber, 2001). However, this approach has limited clinical application due to the need for multiple injections for lasting neuroprotection. Consequently, gene therapy-based strategies have been investigated for their potential to provide more durable neuroprotective effects. Two initial studies demonstrated that a prophylactic intravitreal injection of an AAV vector expressing the BDNF transgene or its receptor TrkB delayed RGC death in rat models of laser-induced ocular hypertension and optic nerve transection, respectively (Martin et al., 2003, Cheng et al., 2002). Although these studies served as proof of concept, pre-symptomatic protective measures have limited clinical relevance, as therapies would be aimed at patients after disease onset occurs. Ren et al. (2012) studied the effectiveness of BDNF expressed from an AAV2 vector in protecting RGCs in a rat model of acute IOP elevation. They found that injecting AAV2-BDNF into the vitreous 6 h post IOP elevation was ineffective at protecting RGC cells in the acute phase of retinal artery occlusion due to the slow onset of transgene expression, but did provide protection in later phases, which more closely resemble the gradual RGC loss observed from chronic IOP elevation in glaucoma. Additionally, supplementing BDNF protein once was effective in compensating for the slow onset of AAV-mediated gene expression and rescued a larger number of RGCs.

While these studies demonstrate the neuroprotective potential of BDNF expression from the retina following viral transduction, this approach comes with potential drawbacks. Firstly, binding of BDNF with its receptor, TrkB, leads to its downregulation and degradation, potentially limiting the effectiveness of sustained delivery (Sommerfeld et al., 2000, Frank et al., 1996). Secondly, overexpression of BDNF may lead to an accumulation of immature proBDNF, and its binding to p75NTR receptors, which are associated with apoptosis and may counteract neuroprotection (Teng et al., 2005). A recent study outlined a novel strategy to circumvent these issues, using an AAV2 vector to deliver a construct expressing both BDNF and TrkB from a single transgene, separated by a sequence encoding a viral 2A peptide linker. The construct contains a modified BDNF transgene lacking its pro- domain and signal peptide sequence which offers two key advantages: enabling expression of both genes within the limited 4.7 kb capacity of the AAV vector and preventing overaccumulation of proBDNF. Controlled by the constitutive cytomegalovirus enhancer/ chicken beta-actin (CAG) promoter, the construct is transcribed as a single RNA, with co-translational cleavage at the viral 2A sequence producing two separate proteins. These mature proteins are subsequently directed to their respective intracellular sites: Trk to the cell surface and BDNF to secretory vesicles for extracellular release (Osborne, Wang, et al., 2018). When injected intravitreally, AAV2 TrkB-2A-mBDNF induced high expression of both transgenes for up to 6 months in mice without causing astrogliosis or microglial activation. In a mouse optic nerve crush model, it provided greater neuroprotection than AAV2-BDNF or AAV2-TrkB alone. Additionally, AAV2 TrkB-2A-mBDNF demonstrated neuroprotective effects in a rat model of laser-induced ocular hypertension (Osborne, Khatib, et al., 2018). Another group developed an approach to generate of a constitutively active form of TrkB by farnesylation of its intracellular domain (F-iTrkB). Intravitreal injection of AAV2-iTrkB was shown to be neuroprotective in a mouse model of normotensive glaucoma (glutamate/aspartate transporter (GLAST) KO mice), a silicon oil model of ocular hypertension, and an optic nerve crush model. Additionally, evidence of axon regeneration was observed in both in the optic nerve crush model and an optic nerve transection model, wherein the lesion was made close to the superior colliculus (Nishijima et al., 2023).

#### 3.2.2 Targeting oxidative stress and mitochondrial dysfunction

Oxidative stress is an imbalance in redox homeostasis, whereby pro-oxidative processes that generate reactive oxygen species (ROS) overcome intrinsic antioxidant defence mechanisms. This leads to DNA, protein and lipid damage in addition to mitochondrial dysfunction which further induces ROS generation. In particular, RGCs are highly susceptible to oxidative damage owing to their high metabolic demand, high lipid and glutamate content, and limited capacity for regeneration (Hurley et al., 2022). Various animal studies suggest that oxidative stress plays a major role in glaucomatous neurodegeneration (Mittag et al., 2000, Ozdemir et al., 2009, Moreno et al., 2004). Additionally, POAG patients display lower antioxidant capacity in the AH and serum relative to cataract controls and this is associated with a higher level of visual field damage, lower RGC numbers, and increased cup-to-disk ratio (Nucci et al., 2013, Tanito et al., 2016, Asano et al., 2017, Abu-Amero et al., 2013). Considering this, the potential of antioxidants as neuroprotective molecules has been explored. For example, rapamycin, a synthetic antioxidant that relieves oxidative stress by inhibiting mTor, has been shown to increase survival of RGCs in a rat chronic hypertension model (Su et al., 2014). As oxidative stress is a common mechanism in glaucomatous neurodegeneration, antioxidant enzymes are attractive gene therapy targets, as such therapies may be beneficial across a range of glaucoma subtypes.

Superoxide dismutase (SOD) is an antioxidant enzyme that catalyses the conversion of the superoxide (O_2_^–^) anion into normal molecular oxygen (O_2_) and hydrogen peroxide (H_2_O_2_) Superoxide is produced as a by-product of oxygen metabolism and, if not regulated, causes many types of cell damage (Culotta, 2001). Jiang et al. (2016) examined the effect of chronic ocular hypertension on retinal oxidative stress and mitochondrial function in a rat laser induced ocular hypertension model. They found that RGC degeneration was associated with a decrease in SOD and catalase activity, and increased levels of malondialdehyde (MDA), the lipid peroxidation decomposition product generated by free radical damage to unsaturated fatty acids. They showed that eyes pre-treated with an intravitreal injection of recombinant AAV expressing SOD2 led to significantly lower levels of MDA accumulation and reduced RGC degeneration through a mitochondria-related pathway. However, MDA content was still higher than baseline even after administration of AAV-SOD2, indicating that the treatment did not eliminate oxidative stress and would not provide lasting RGC protection.

Nuclear factor erythroid 2-like 2 (NRF2) is a transcription factor that plays a crucial role in the response of cells against oxidative insults. NRF2 is activated by ROS, leading to the upregulation of antioxidant responsive elements- associated genes and the expression of several antioxidant proteins such as glutathione peroxidase, glutathione reductase, thioredoxin, and SODs 1, 2, and 3 (Panieri et al., 2022). Intravitreal injection of AAV2-NRF2 has been shown to be protective against RGC degeneration in a mouse optic nerve crush model (Xiong et al., 2015). In a later study, Fujita et al. (2017) aimed to optimise this strategy to only target damaged RGCs. They highlighted that many ocular gene therapy approaches, including that of Xiong et al. (2015) use constitutively active promoters to drive expression of therapeutic transgenes. However, such promoters result in expression of the therapeutic gene in both injured and healthy cells, which could induce stress responses in healthy cells by creating a non-physiological environment. They identified the monocyte chemoattractant protein 1 (Mcp-1) promoter, which is involved in leukocyte recruitment, as being able to drive transgene expression specifically in injured RGC cells (Toney et al., 2000). They showed that Mcp-1driven NRF2 expression showed equivalent efficacy in protecting RGCs in a mouse optic nerve crush model to that driven by the CMV promoter used by Xiong et al. (2015). However, CMV mediated NRF2 expression induced cellular stress and death in healthy RGCs, which were significantly reduced by utilising the Mcp-1 promoter.

#### 3.2.3 Targeting Wallerian degeneration

Wallerian degeneration is a programmed axonal death process that occurs after a nerve fibre is cut or damaged, leading to the degeneration of the axon segment separated from the neuron's cell body. This process is closely linked to reduced levels of axonal nicotinamide adenine dinucleotide (NAD + ) (Figley and DiAntonio, 2020). Maintaining NAD + levels is essential to prevent axonal degeneration (Wang et al., 2005, Wang et al., 2015). Nicotinamide mononucleotide adenylyltransferase 2 (NMNAT2) and sterile alpha and TIR motif containing 1 (SARM1), are crucial mediators of programmed axonal death. NMNAT2 is responsible for NAD + biosynthesis; its depletion can induce Wallerian degeneration even in uninjured axons (Gilley and Coleman, 2010). Conversely, SARM1, with its intrinsic NAD + hydrolase activity, drives axonal degeneration by depleting NAD + rapidly after axonal injury. The subsequent NAD + depletion leads to decreased ATP levels and energy deficit, triggering cytoskeletal disintegration and loss of axonal membrane integrity (Conforti, Gilley, and Coleman, 2014). In glaucoma, axon degeneration is an early feature of glaucomatous optic neuropathy (Howell et al., 2007, Beirowski et al., 2008). The wallerian degeneration slow (Wlds) allele, which encodes a mutant chimeric NMNAT1 localized to axons, has shown protective effects in both the optic nerve crush model and DBA/2J mice (Howell et al., 2007, Beirowski et al., 2008). Additionally, vitamin B3 (an NAD + precursor) administration has also demonstrated protective effects against axonal degeneration in DBA/2J mice (Williams et al., 2017).

In recent years, numerous gene therapy-based strategies targeting Wallerian degeneration in glaucoma have been developed. One such strategy involves inducing the overexpression of NMNATs in retinal ganglion cells (RGCs) to maintain adequate NAD + levels and prevent axonal degeneration. For example, Williams et al. (2017) injected an AAV2.2 vector expressing murine *Nmnat1*, driven by a CMV promoter, into the vitreous of DBA/2J mice at 5.5 months of age. By 12 months, the treatment resulted in robust transgene expression, effectively preventing RGC axon and soma loss, preserving axoplasmic transport, and maintaining electrical activity in the RGCs. In another study, Fang et al. (2022) used RiboTag RNA-seq to characterise the translatome of RGCs in mice with silicone oil-induced ocular hypertension. They found that the expression of NMNAT2, but not NMNAT1 or NMNAT3, was significantly reduced relative to control eyes. Based on these findings, they designed a gene therapy approach that involved intravitreal injection of an AAV2 vector expressing a long half-life mutant NMNAT2 lacking exon 6, driven by an RGC-specific mouse γ-synuclein promoter (mSncg). This therapy provided significant neuroprotection of both RGC soma and axons, and preserved visual function in both a mouse optic nerve crush model and the silicone oil-induced ocular hypertension model.

To maintain NAD + levels and prevent axonal degeneration, another strategy is to disrupt the activity of the pro-degenerative enzyme SARM1. Liu et al. (2023) explored the effects of disrupting Sarm1 on glaucomatous neurodegeneration using two gene-based approaches: administering antisense oligonucleotides (ASO) against Sarm1 and CRISPR/Cas9-mediated knockdown of Sarm1 through co-transfection with AAV2 vectors containing Cas9 and SARM1 gRNAs under the control of an mSncg promoter. The neuroprotective capabilities of these approaches were compared to germline knockout of Sarm1 in both an optic nerve crush model and a silicone-oil induced ocular hypertension model. In the optic nerve crush model, all three methods of Sarm1 disruption preserved optic nerve axons but did not prevent RGC soma death. In the silicone-oil induced ocular hypertension model, both RGC somata and axons were preserved. However, unlike germline knockout of Sarm1, neither ASO nor CRISPR-mediated disruption improved visual acuity in either model, indicating that these methods are less effective in inhibiting SARM1 than germline deletion.

## 4 Future directions and research priorities

### 4.1 Improving viral vector efficiency

An important consideration in gene therapy is the selection of an appropriate vector for gene delivery. Vectors utilised in gene therapy can be broadly classed into viral and non-viral delivery systems. Although this review offers a brief discussion of vectors in gene therapy for glaucoma, the topic has been explored in greater depth elsewhere (Amador et al., 2022, Hakim et al., 2023). Most ocular gene therapy clinical trials to date have used viral vectors. For example, Luxturna (Spark Therapeutics), the first ocular gene therapy approved by the FDA in 2017 for treating Leber’s congenital amaurosis type 2, introduces a gene encoding the retinoid isomerohydrolase enzyme using an AAV2 vector (Fischer, 2017). In glaucoma, the appropriate vector depends on the tissue being targeted. Most gene therapies aimed at reducing IOP target the TM, while neuroprotection-focussed therapies target RGCs. Thus, many studies have focussed on determining the ideal vector for delivering genetic cargo to these tissues.

The natural flow of AH directs injections of recombinant viruses in the anterior chamber to the TM, making it an ideal target for gene therapy. Adenoviruses are capable of transducing the TM in mouse, monkey and human eyes (Borrás et al., 2001, Budenz et al., 1995, Borrás et al., 1999). However, they are highly immunogenic and do not provide long lasting gene expression (Borrás, Tamm, and Zigler 1996). Single-stranded (ss) AAVs are favoured for gene therapies targeting the posterior segment due to their durable expression and low immunogenicity. However, their ability to transduce TM cells is limited. This limitation arises from the host's downregulation of DNA replication, which hinders the conversion of the ssAAV genome into double-stranded DNA (Borrás et al., 2006). To circumvent this, studies have employed self-complementary AAVs (scAAVs) that contain both the sense and antisense cDNA strands of the transgene. In particular, scAAV2 has been demonstrated to have a high transduction efficiency in the TM of rodent and primate models (Buie et al., 2010, Bogner et al., 2015). Recent efforts have been made to further characterise and optimise scAAVs as IOP lowering gene therapy vectors. For example, one study investigated whether an ocular hypertensive environment would influence the tropism of scAAVs, as previous research had only tested them under normotensive conditions. The transduction efficiencies of scAAVs 2, 5, and 8, each packaged with EGFP, were assessed in the anterior segment structures of rats with ocular hypertension induced by circumlimbal suturing. The results showed that scAAVs 2 and 5 could efficiently transduce the TM, with scAAV5 displaying higher specificity. Notably, the transduction efficiencies of these vectors increased under ocular hypertensive conditions due to the enhanced expression of their cognate receptors (Lee et al., 2019).

The efficiency and tropism of AAVs is highly species specific. Thus, findings from rodents do not necessarily translate to humans and it is important that vector efficiency is tested in the human relevant tissue. Another study investigated the transduction efficiencies of seven scAAV serotypes (1, 2, 2.5, 5, 6, 8, and 9) in primary human TM cells. Of these, scAAV2 was found to have the highest transduction efficiency, showing a 3.1x higher transgene expression than the next best serotype (scAAV2.5). It was further shown that scAAV with a tyrosine triple mutant capsid exhibited a 3.5x higher transduction efficiency than non-mutated scAAV2, suggesting that this may be the optimal vector to deliver genes to the TM and attempt to control elevated IOP (Rodriguez-Estevez, Asokan, and Borrás, 2020).

As AAV2 is the gold standard serotype for posterior segment gene therapies and effectively targets RGCs when injected intravitreally in rodents (Han et al., 2020). However, rodent eyes are significantly different to primate and human eyes, as they lack a macula. The macula in humans, in addition to having a high photoreceptor density, can have up to 10 rows of RGCs and has a correspondingly thick nerve fibre layer, in contrast to mice which have a single RGC layer throughout the retina. In addition to this, humans have a highly developed inner limiting membrane (ILM). Thus, rodent RGCs are significantly easier to transduce (Starr and Chen, 2023). Indeed, intravitreal injection of AAV2 into the macaque eye has been shown to transduce a small population of RGCs in the fovea, but exhibits limited transduction beyond this (Yin et al., 2011).

This is a major reason that Lenadogene nolparvovec, a gene therapy for Leber’s congenital optic neuropathy that utilises a recombinant AAV2/2 vector injected intravitreally to deliver a human wild-type ND4 transgene to RGCs, while generally safe, has shown only marginal benefit (Wan et al., 2016, Guy et al., 2017, Newman et al., 2022). RGC transduction efficiency has been shown to be improved in primates by performing vitrectomy or peeling back the ILM before intravitreal injection (Takahashi et al., 2017, Tshilenge et al., 2016). However, the adverse effects associated with such invasive procedures would need to be investigated before such approaches would be clinically applicable. Novel AAV capsids may provide better transduction efficiencies. For example, both AAV7m8 and AAV8BP2 have been shown to transduce RGCs in non-human primates, with AAV7m8 transducing parafoveal RGCs at high viral titres (Ramachandran et al., 2016).

An alternative strategy to circumvent the ILM involves delivering an AAV to RGCs via retrograde transport from their axon terminals. A variant of AAV2, recombinant AAV2-retro (rAAV2-retro), has been developed to increase its capacity for retrograde transport (Tervo et al., 2016). In a recent study, it was shown that when rAAV2-retro carrying fluorescent reporter genes was injected into the superior colliculus of rats, this led to widespread transduction of RGCs that lasted up to 375 days (Zheng et al., 2024). This highlights the potential utility of injecting rAAV-retro into the lateral geniculate nucleus, the primary projection site for RGCs in primates and humans, for RGC gene therapy. However the authors note that this approach comes with the disadvantage of requiring a neurosurgical procedure and would transduce both eyes simultaneously, which would make it impossible to compare a treated and control eye, a preferred comparison for clinical trials (Zheng et al., 2024).

### 4.2 Improving transgene regulation

Another crucial factor in gene therapy is the promoter that drives the expression of the therapeutic transgene. Due to the low transduction efficiency of AAV2 vectors in human RGCs, many studies have focused on improving transgene expression using RGC-specific promoters. One prominent example is the gamma synuclein (SNCG) promoter, which is specifically and ubiquitously expressed in RGCs of both rodents and humans (Surgucheva et al., 2008). A recent study demonstrated that a transgene encoding EGFP, delivered via an AAV2 vector with a mouse Sncg (mSncg) promoter, resulted in better RGC-specific gene expression in mice and human TM cells compared to the commonly used CAG promoter. Additionally, it was demonstrated that a truncated form of the mSncg promoter, while slightly less RGC specific, could be packaged into an AAV2 vector to drive expression of a CRISPR/Cas9 system to knockdown pro degenerative genes *Ddit3* and *Sarm1*, providing significant neuroprotection in an optic nerve crush model (Wang et al., 2020).

Another study has used a human *SNCG* (*hSNCG*) promoter to drive expression of channelrhodopsin in macaque RGCs (Chaffiol et al., 2017). Interestingly, Wang et al. (2020) found that m*Sncg* led to higher transgene activation than *hSNCG*, even in human RGC cells, indicating that *mSncg* may be an optimal promoter for driving transgene exporession in human gene therapy.

As AAV vectors have a small packaging capacity, further studies have aimed to short promoter elements to maximise space for delivering large cargo. One study highlighted the human synapsin (hSYN) promoter as being able to drive RGC specific transgene expression in mice. However this was not observed in post-mortem human retinal explants, which may be attributable to the low viability of neurons in post-mortem tissue and the presence of the INL (Nieuwenhuis et al., 2023). As mentioned previously, there are likely populations of both healthy and injured RGCs dispersed throughout the glaucomatous retina. RGC specific promoters, like constitutive promoters, come with the drawback of targeting both healthy and injured RGC cells, which can induce a stress response in healthy cells by creating a non-physiological environment. Fujita et al. (2017) demonstrated that transgene expression driven by the Mcp-1 promoter can target stressed RGC cells specifically and shows equivalent transgene expression to the CMV promoter, while reducing stress and death in healthy RGCs. Advancing the development of such targeted promoters could significantly enhance the efficiency of gene therapies for neuroprotection in humans.

### 4.3 Development of non-viral vectors

In recent years, non-viral vectors have come to prominence as an alternative to viral vectors. Non-viral vectors include a range of synthetic and natural systems to transport genetic cargo into cells without using viruses. These include lipids, micelles, metal atom substrates, polymers, and peptides. Non-viral vectors present a safer and more flexible alternative to viral vector mediated gene therapy, as they have a larger packaging capacity and are less immunogenic. However, they face challenges such as lower delivery efficiency and more transient gene expression compared to viral vectors (Hakim et al., 2023). While a detailed discussion of non-viral vectors is beyond the scope of this review, several of the IOP-lowering gene therapy strategies outlined here have utilised non-viral vector systems effectively. For example Dillinger et al. (2018) utilised layer-by-layer coated nanoparticles, with a hyaluronic acid (HA)-based outer layer to deliver CTGF-siRNA to the TM. In addition to circumventing the issue of immunogenicity of viral vectors, this approach has the advantage of targeting CD44, which is upregulated in the glaucomatous anterior chamber, thus providing specificity towards tissue with pathological expression changes. Additionally, Tan et al. (2021) utilised a polydopamine–polyethylenimine nanoparticle vector to deliver an ECM-regulating miRNA to TM cells, which was shown to enhance its stability while causing minimal cytotoxicity. A hyperbranched cationic glycogen derivative vector has also been used to deliver IκBα-siRNA intracamerally in normotensive rats, leading to a transient reduction in IOP (Zeng et al., 2020). Despite these positive developments, it is crucial to optimise nanoparticle vectors to prevent accumulation in the outflow pathways, which could lead to blockages or tissue damage. Developing non-viral vectors for retinal ganglion cells (RGCs) remains more challenging due to the inner limiting membrane (ILM) and the absence of a natural flow pattern towards the target tissue, unlike the flow of AH in the anterior chamber. Nonetheless, as novel non-viral delivery mechanisms continue to be developed, they may provide a superior alternative to viral vectors in the future.

## 5 Conclusion

Gene therapy holds significant promise in glaucoma treatment, offering potential long-term solutions by targeting the disease's underlying causes at a molecular level. Current treatments primarily focus on reducing intraocular pressure (IOP) through medications or surgery, often facing limitations in efficacy and patient adherence. Gene therapy strategies for glaucoma can be broadly classed into those aimed at reducing IOP and those aimed at providing neuroprotection to RGCs. As most glaucoma is a multifactorial disease, gene therapies aim to target common pathways underlying these hallmarks of disease. In the case of reducing IOP, this can be achieved by increasing ECM turnover or reducing AH production. Neuroprotective strategies target common RGC degenerative pathways such as neurotrophic factor deprivation, oxidative stress, and Wallerian degeneration. While many positive strides have been made in these areas, particularly in rodent model systems, challenges remain in optimising delivery systems for specificity, efficacy, and safety. Continued research in non-human primates, which have eyes more anatomically similar to humans, will be crucial for translating these approaches into clinically useful therapies.

## CRediT authorship contribution statement

**Jeff Henderson:** Writing – original draft, Conceptualization. **Jeffrey O’Callaghan:** Writing – review & editing, Writing – original draft, Conceptualization. **Matthew Campbell:** Writing – review & editing, Conceptualization.

## Data Availability

No data was used for the research described in the article.
